# Vertical Profiles of Bacteria in the Tropical and Subarctic Oceans Revealed by Pyrosequencing

**DOI:** 10.1371/journal.pone.0079423

**Published:** 2013-11-13

**Authors:** Hongmei Jing, Xiaomin Xia, Koji Suzuki, Hongbin Liu

**Affiliations:** 1 Division of Life Science, The Hong Kong University of Science and Technology, Clear Water Bay, Kowloon, Hong Kong SAR, China; 2 Sanya Institute of Deep-Sea Science and Engineering, Chinese Academy of Sciences, Sanya, China; 3 Faculty of Environmental Earth Science, Hokkaido University, Sapporo, Japan; Wageningen University, Netherlands

## Abstract

Community composition of Bacteria in the surface and deep water layers were examined at three oceanic sites in the Pacific Ocean separated by great distance, i.e., the South China Sea (SCS) in the western tropical Pacific, the Costa Rica Dome (CRD) in the eastern tropical Pacific and the western subarctic North Pacific (SNP), using high throughput DNA pyrosequencing of the 16S rRNA gene. Bioinformatic analysis rendered a total of 143600 high quality sequences with an average 11967 sequences per sample and mean read length of 449 bp. Phylogenetic analysis showed that *Proteobacteria* dominated in all shallow and deep waters, with *Alphaproteobacteria* and *Gammaproteobacteria* the two most abundant components, and SAR11 the most abundant group at family level in all regions. *Cyanobacteria* occurred mainly in the surface euphotic layer, and the majority of them in the tropical waters belonged to the GpIIa family including *Prochlorococcus* and *Synechococcus*, whilst those associated with Cryptophytes and diatoms were common in the subarctic waters. In general, species richness (Chao1) and diversity (Shannon index *H*′) were higher for the bacterial communities in the intermediate water layers than for those in surface and deep waters. Both NMDS plot and UPGMA clustering demonstrated that bacterial community composition in the deep waters (500 m ∼2000 m) of the three oceanic regions shared a high similarity and were distinct from those in the upper waters (5 m ∼100 m). Our study indicates that bacterial community composition in the DOC-poor deep water in both tropical and subarctic regions were rather stable, contrasting to those in the surface water layers, which could be strongly affected by the fluctuations of environmental factors.

## Introduction

Bacteria are important components of marine ecosystems and play a critical role in the biogeochemical cycles [1]. They comprise up to 70 % and 75 % of the total biomass in surface [2] and deep waters [3], respectively. Although bacteria are ubiquitous and abundant in marine ecosystems, relatively little is known about their diversity and composition, which is usually affected by various environmental parameters. The interaction between bacterial assemblages with their living environments shapes the bacterial community structure and affects the function of various bacterial groups. One of the most relevant parameters in aquatic systems is the depth through the water columns, which harbor not only dramatic physicochemical gradients but also marked microbial community shifts. Microbial studies on different depths in the Northeast Pacific and the subtropical and temperate Atlantic Oceans [4], [5] and Antarctic Polar Front [6] revealed that numerous novel prokaryotic lineages exist in the deep sea, suggesting that they may play important ecological roles in the deep waters. However, until now the variability of bacterial communities through the depth of water column and among different parts of the global ocean is still only poorly known.

Methodological constraints have limited the progress in the field of microbial diversity research, especially for microbial groups with high diversity but low abundance. Recently, the application of next generation sequencing technology, such as 454 pyrosequencing, has allowed us sequencing reads orders of magnitude greater than conventional sequencing approaches [4], [7], and resulted to the revealing of up to 106–109 marine bacterial taxa [8]. This deep sequencing method is capable of detecting both the most abundant community members and rare species which could not be retrieved by traditional cultivation, cloning and sequencing methods [8], therefore enables statistically robust assessments of community structures and dynamics and provides a more realistic/exhaustive picture of the microbial community.

Pyrosequencing has been applied to investigate different microbial communities in various marine ecosystems, such as the North Pacific Ocean [4], [9], [10], [11], [12], Mediterranean Sea [13], Baltic Sea [14], [15], Atlantic Ocean [16], [17], [18] and Arctic Ocean [19]. Lots of unique prokaryotic species together with unexpectedly high diversity of Bacteria and Archaea were uncovered in deep-sea pelagic and benthic communities [10], [16]; and planktonic Bacteria in the upper ocean were found much less diverse than those in the deep sea [20]. However, vertical bacterial community profiles in the same ocean but thousand kilometers apart have not been extensively investigated, and whether similar community shifts patterns are congruent across the ocean basins remains uninvestigated.

In our study, water samples were collected from three geographically separated oceanic sites located in both tropical and subarctic North Pacific Ocean, including the western tropical North Pacific characterized by permanent stratification and oligotrophic conditions, the eastern tropical North Pacific featured an shallow thermocline depth caused by upwelling and a permanent oxygen deficient zone between 400 and 700 m, and a subarctic site located in the Oyashio region where a spring phytoplankton bloom occurs annually. In each region, the microbial communities from four different depths covering from surface photic zone to deep water, along the steep gradients of light, temperature, pressure, macronutrient and trace metal concentrations, were examined by high throughput pyrosequencing based on 16S rRNA gene. The objectives of this study are to investigate the vertical profiles of microbial communities across the broad depth scale and to compare the microbial community structures in the tropical and subarctic North Pacific Ocean.

## Materials and Methods

### Sample collection and DNA extraction

Seawater samples were collected from 3 separate sites in tropical and subarctic North Pacific Ocean: 1) the Southeast Asia Time-Series Study station (SEATS) (18° 30′N, 115° 5′E), which is located at the deep central basin of the South China Sea (SCS), during the GOE-2 cruise on board the research vessel *Dongfanghong#2* in August 2007, 2) the subarctic northwest Pacific Ocean (SNP) (41°30′N, 144°E) during an R/V *Taisei Maru* (JAMSTEC) cruise in May 2011 when a spring phytoplankton bloom occurred, and 3) the Costa Rica Dome (CRD) in eastern tropical Pacific (9° 2.106′N, 90° 33.741′W) during the FLUZiE cruise in July 2010. No specific permissions were required for sampling in these locations, and our study did not involve endangered or protected species. Water from four different depths (5, 100, 500 and 2000 m for SCS, 10, 100, 500 and 1500 m for SNP, and 40, 200, 500 and 2000 m for CRD), was collected using a CTD carousel water sampler with Go-Flo or X-Niskin bottles (General Oceanic). Between 1 and 2.4 liters of seawater were filtered onto 0.22 µm pore-size polycarbonate membranes (47 mm diameter, Millipore) on board. The membranes were stored at −80°C until DNA extraction on land.

Total genomic DNAs were recovered from biomass collected with the 0.22 µm filters following the instruction of PureLink™ Genomic DNA Mini Kit (Invitrogen). Extracted DNAs were precipitated with isopropanol and then stored at −80°C.

### PCR amplification and 454 pyrosequencing

The environmental DNAs were amplified with universal bacterial primers 16S-341F (5′-CCTAYGGGRBGCASCAG-3′) and 16S-806R (5′-GGACTACNNGGGTACTAAT-3′) flanking the V3 and V4 regions of 16S gene [21] by using FastStart High Fidelity PCR system, dNTPack (Roche). Different MID sequences for pyrosequencing were synthesized together with forward primer. PCR was carried out with a Peltier Thermal Cycler (Bio-Rad) with the following cycles: 95 oC for 2 mins; 35 cycles of 95 oC for 30 sec, 55 oC for 30 sec, 72 oC for 40 sec; final extension at 72 oC for 7 mins. Triplicate PCR products for each sample were combined and subsequently purified by illustra™ GFX™ PCR DNA and Gel Band Purification kit (GE Healthcare). An amplicon library was constructed and emPCR was conducted according to the instructions of Rapid library preparation (Roche, 454 Life Science). DNA beads were successfully deposited onto the PicoTiterPlate and sequenced by a GS Junior system (Roche, 454 Life Science).

### Post-run Sequence analysis

All forward-oriented reads generated from this study were separated according to their specific multiplex identifiers (MIDs), and quality control was performed using the RDP pyrosequencing pipeline. For example, reads were flagged as low quality when they had <300 bp in length, the start of the sequence did not exactly match a primer sequence, or one ambiguous base (N) was presented in the sequence [22]. Chimeras were detected with the Chimera Slayer algorithm implemented in Mothur software package using the 16S rDNA data sets as references. After above filtration, the remaining reads were further analyzed with Mothur for alignment, distance calculation and classification [23]. Taxonomic identification of each read was carried out with Mothur against the Silva bacteria no-gap reference database at a cutoff value of 60.

The richness estimator (Chao1), diversity (Shannon index *H*′), coverage (Chao) and operational taxonomic unit (OTU) numbers were calculated at the cutoff level of 3% using Mothur's *summary.single* routine. Rarefaction curves were calculated using *rarefaction.single* with 10000 iterations. Nonmetric Multidimensional Scaling (NMDS) analysis was applied to compare the bacterial community composition among all the samples using Primer 5 [24] based on the classification of different phylogenetic groups. To estimate similarity among samples, hierarchical cluster analysis was also conducted based on a matrix of different OTUs and their abundance in each sample using Bray-Curtis similarity calculated from Mothur and a dendrogram inferred with the unweighted pair-group average algorithm (UPGMA). In addition, Venn diagrams at 3% sequence dissimilarity were generated using Mothur to show both unique and shared OTUs in each water sample. All sequences obtained from this study have been deposited in the National Center for Biotechnology Information (NCBI) Sequence Read Archive (SRA) under accession number SRX286892.

## Results

### Sequencing statistics and diversity estimates

Pyrosequencing generated 172103 raw sequence reads and a total 143600 reads (46673 reads for SNP, 52119 reads for SCS and 44808 reads for CRD) were remained after filtering out low-quality reads according to the applied criteria described in methodology (Table 1). On average, 11668 reads per sample and 451 bp per read were obtained for samples from SNP, 13030 reads per sample and 448 bp per read were retrieved for samples from SCS, and 11202 reads per sample and 448 bp per read were retrieved for those from CRD. Using 3% sequence cutoff value, a total of 5097, 7199 and 4717 OTUs were obtained from SNP, SCS and CRD, respectively (Table 1). In term of unique OTUs, three different patterns were observed at three regions – maximum numbers in the intermediate water layers (100 m and 500 m) in SNP, increasing with depth in SCS, and no clear trend in CRD. Comparatively, the number of OTUs, species richness and diversity in SCS were relatively higher than those in the other two regions. In addition, coverage for all the samples at the three stations ranged from 84 % to 94 % (Table 1).

**Table 1:** Sequencing information and diversity estimates for water samples collected from four different depths at 3 sites in subarctic and tropical North Pacific Ocean obtained by 454 pyrosequencing.

Station	Depth(m)	No. of Sequences*	Average length (bp)	OTUs		Richness	Diversity	Coverage
				Total	Unique	Chao1	Shannon index (*H*′)	Chao (%)
**Subarctic North Pacific** (total reads = 46673)								
	10	14638	452	796	503	2387	4.28	93.7
	100	12449	452	1544	1120	5475	4.85	86.8
	500	12423	450	1841	1268	5684	4.71	84.0
	1500	7163	451	916	606	2360	4.14	88.0
**South China Sea** (total reads = 52119)								
	5	11523	446	1258	943	5618	4.31	87.4
	100	13911	447	1923	1311	6989	5.07	85.4
	500	12789	451	1969	1344	5231	5.05	85.4
	2000	13896	450	2049	1288	5221	5.40	85.8
**Costa Rica Dome** (total reads = 44808)								
	40	12997	446	1068	652	2664	4.17	92.1
	200	10784	448	1444	901	3671	4.93	87.1
	500	9128	452	1103	651	2648	4.99	89.2
	2000	11899	447	1102	788	2986	4.68	93.5

* Trimmed reads that passed quality control; Total number of OTUs, species richness estimator (Chao1), diversity (Shannon index *H*′), coverage (Chao) were calculated at 97% similarity level.

**Figure 1:**
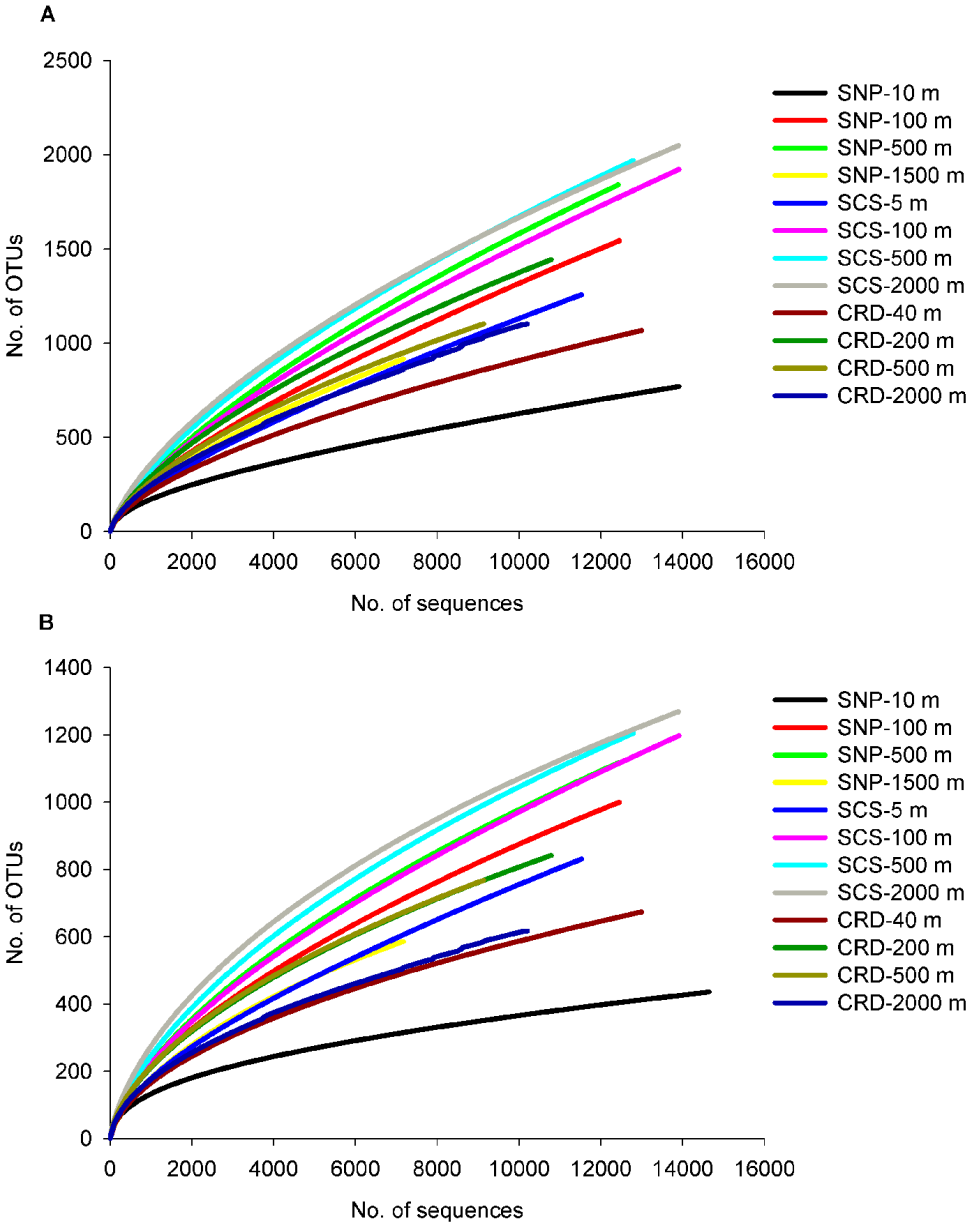
Rarefaction curves for total OTUs. Curves were generated for OTUs at species level (a) with cutoff value of 3 % and at genus level (b) with cutoff value of 5 % based on sequences obtained by pyrosequencing from water samples collected from four different depths in subarctic North Pacific Ocean (SNP), tropical South China Sea (SCS) and Costa Rica Dome (CRD).

Along the vertical profiles, similar patterns of richness (Chao1) and diversity (Shannon index *H*′) were shown in all the three oceanic regions: higher in the intermediate waters (100 m and 500 m) than in the surface and deep waters. This pattern was also reflected by the rarefaction curves with both cutoff values of 3% on the species level (Fig. 1a) and 5% on the genera level (Fig. 1b): curves for surface and deep waters became flat earlier than those from intermediate waters. Generally, the intermediate water harbors higher bacterial diversity than the surface and deep waters (Table 1), and it requires more sampling efforts in order to adequately assess the bacterial community composition in the intermediate water.

### Microbial community structure

**Figure 2:**
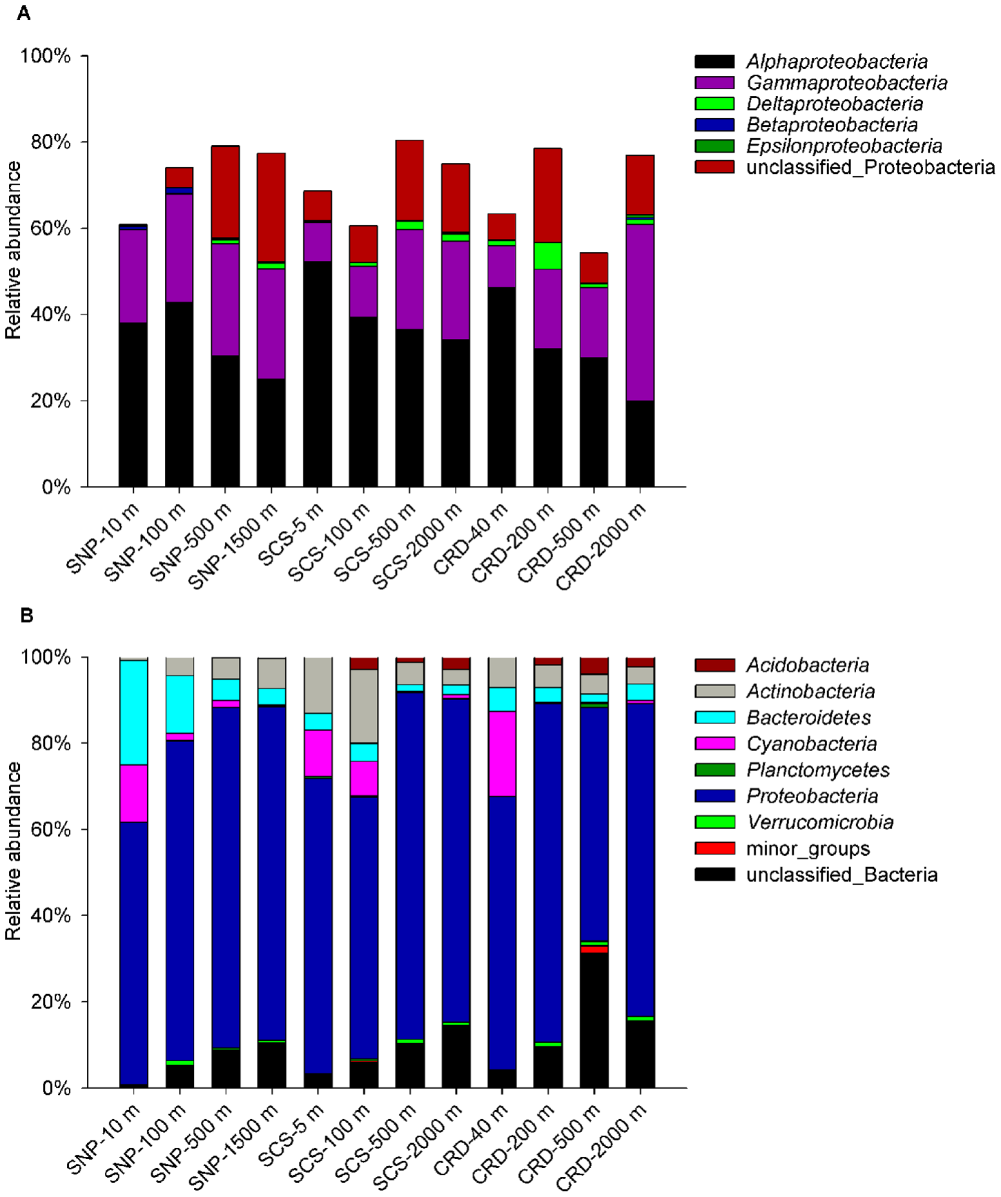
Relative abundance of the bacterial taxonomic groups. Mothur was applied to identify sequences on the phylum level (a) and on the class level of *Proteobacteria* (b) for water samples collected from four different depths in subarctic North Pacific Ocean (SNP), tropical South China Sea (SCS) and Costa Rica Dome (CRD).

In total, seven bacterial phyla were identified from all samples and *Proteobacteria* was the major phylum existed in different water bodies in the three oceanic regions, counting for more than 60% of the whole bacterial communities (Fig. 2a). Phyla of *Actinobacteria* and *Bacteroidetes* were also detected in each sample but with much less abundance, whilst phyla of *Acidobacteria*, *Cyanobacteria* and *Verruccomicrobia* were found only in some samples and phylum of *Planctomyces* was only detected in the sample of CRD-500 m, which is located in the oxygen minimum zone (OMZ). *Cyanobacteria* was more abundant in the surface waters (11∼20 %) of the three oceanic regions.

Five *Proteobacteria* classes were identified from all samples (Fig. 2b). *Alphaproteobacteria* was the most abundant class, particularly in the surface waters of all three regions (38 % ∼52 %) and was slightly more abundant in the tropical stations. *Gammaproteobacteria* was the second major group, but their abundance was much lower (9∼28 %) than *Alphaproteobacteria*. One general distribution pattern was the decrease of relative abundance of *Alphaproteobacteria* and increase of relative abundance of *Gammaproteobacteria* with water depth; and the abundance of these two groups became comparable in deep waters. In addition, *Betaproteobacteria*, *Deltaproteobacteria* and *Epsilonproteobacteria* were also found in different water bodies but they accounted for only small portions.

**Figure 3:**
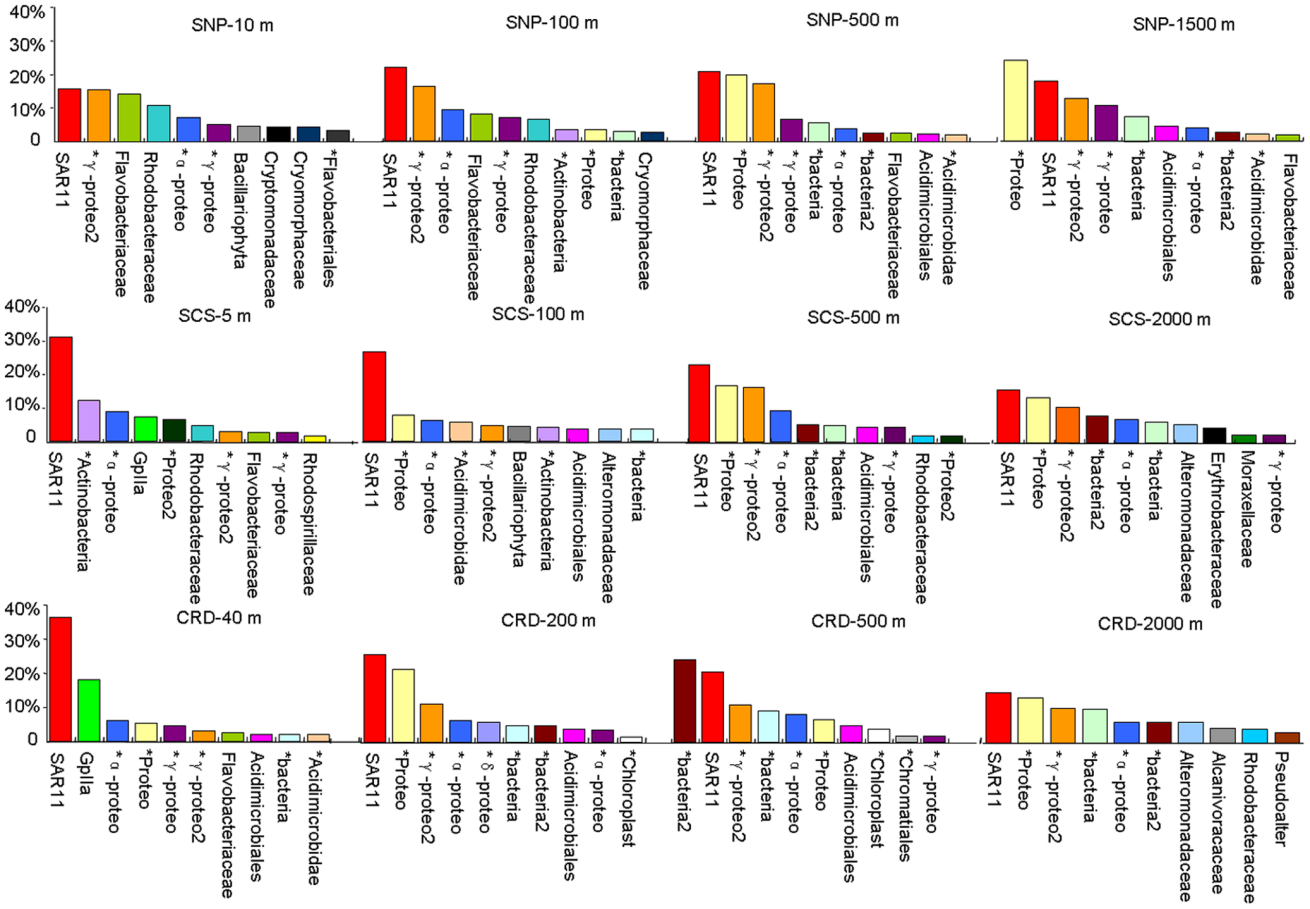
The 10 most abundant OTUs. Relative abundance and affiliation of the 10 most abundant OTUs on the family level for water samples collected from four different depths in subarctic North Pacific Ocean (SNP), tropical South China Sea (SCS) and Costa Rica Dome (CRD). *unclassified. *Proteo* represents *Proteobacteria; Pseudoalter* represents *Pseudoalteromonadaceae*.

The top 10 most abundant OTUs at the family levels in all the samples demonstrated a clear shift among the geographic locations and depths (Fig. 3). SAR11 and other *Proteobacteria* were consistently the most or second most abundant OTU throughout the water column of the three regions. It is noticeable that the top three most abundant OTUs in aphotic layer (deeper than 200 m) of all three sites were the same, namely SAR11, unclassified *Proteobacteria* and *Gammaproteobacteria*2, except CRD-500 m which saw the OTU of an unclassified bacteria took the first place. On the contrary, the composition of the most abundant OTUs in the euphotic layer was more variable. *Cyanobacteria*, GpIIa family in the tropical waters, and chloroplasts associated with *Bacillariophyta* and *Cryptomonadaceae* in the subarctic waters, was among the most abundant OTUs in the surface layers. In addition, certain OTUs, such as *Alcanivoracaceae*, *Erythrobacteraceae*, *Moraxellaceae* and *Pseudoalteromonadaceae*, appeared only in the deepest water layer (2000 m).

### Community similarity

**Figure 4:**
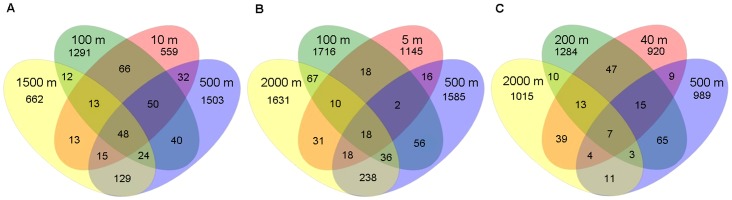
Similarity in bacterial composition among different water depths. Venn diagrams representing the overlap of OTUs (at 3 % sequence cutoff value) among water samples collected from four different depths in subarctic North Pacific Ocean (SNP), tropical South China Sea (SCS) and Costa Rica Dome (CRD).

**Figure 5:**
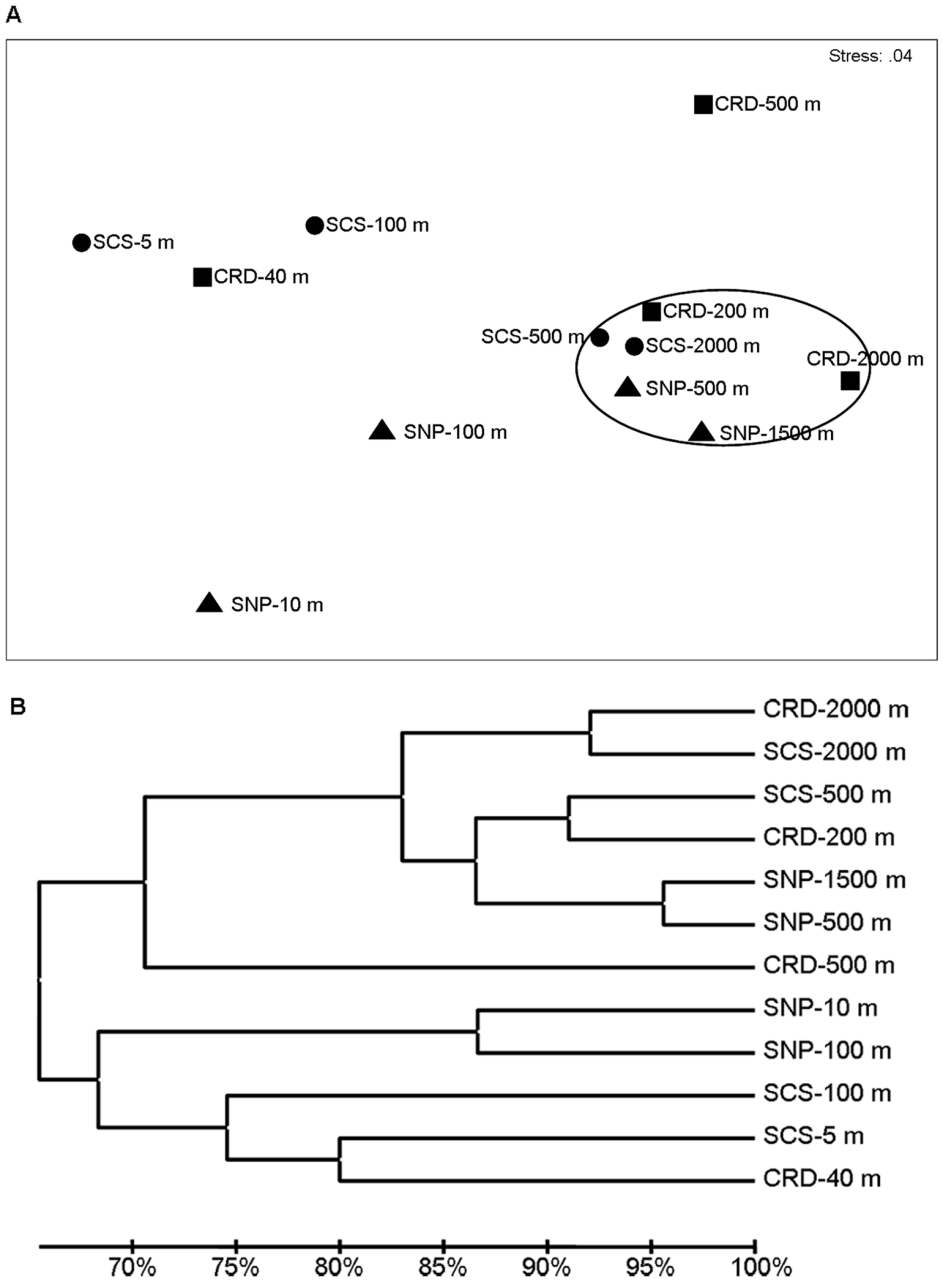
Grouping of bacterial communities among different geographic locations and water depths in the Pacific Ocean. Relationship among bacterial communities in water samples collected from four different depths of 3 geographically separated oceanic sites in the Pacific Ocean demonstrated by the NMDS plot (a) and UPGMA dendrogram (b). SNP – subarctic western North Pacific Ocean, SCS – South China Sea in tropical northwestern Pacific, CRD – Costa Rica Dome in tropical northeastern Pacific.

Venn diagrams were plotted to show the similarities among different microbial communities at different depths in the same oceanic region in term of the overlapping of OTUs (3% sequence cutoff value) (Fig. 4). In the subarctic SNP waters, 48 OTUs were shared by all the water samples; 216 OTUs were detected at both 500 m and 1500 m; 177 OTUs were found at both 10 m and 100 m; and 89 OTUs were present only at 10 m and 1500 m. The number of specific OTUs occurred in the intermediate water depths (100 m and 500 m) was much higher than in the surface and deep waters. Similarly, the highest number of common OTUs (310 OTUs) was found at 500 m and 2000 m; whilst 77 OTUs was found in the water of 5 m and 2000 m in the tropical SCS waters and only 18 OTUs was detected from all the depths. In contrast, the common OTUs shared by the 500 m and 2000 m waters were very low (25 OTUs) in the tropical CRD waters and only seven OTUs existed in all the depths. To compare the difference of bacterial community composition among the three oceanic regions, nonmetric multidimensional scaling (NMDS) plot was further performed. Samples from deep layers (500 m∼2000 m) in different oceanic regions were closely clustered together and clearly separated from samples from shallow layers, indicating high similarity among the bacterial communities in deep oceanic waters despite magnificent distance apart (Fig. 5a). One noticeable exception is the CRD-500 m, where a permanent oxygen deficiency occurred. In addition, UPGMA clustering dendrogram supported the result of the NMDS analysis by revealing that bacterial communities in the deep waters (500 m ∼2000 m) of all three oceanic regions shared higher similarity (>80 %) than those from other depths, except the CRD-500 m, which formed a separate cluster and was distinct from other deep waters due to its permanent OMZ environment (Fig. 5b). On the other hand, bacterial communities in the upper layers of the same oceanic region clustered together but were distinct based on oceanic regions, and SCS and CRD, which are both located in the tropical regions, clustered closer to each other.

## Discussion

Bacteria play a central role in energy and matter fluxes in the sea and their metabolic activities are recognized as major drivers of the biogeochemical processes in the oceans, yet the genetic diversity and variability of their community composition among various oceanic realms are still poorly understood. It has been a long-standing interest in the field of marine microbiology to study how microbial assemblages vary in space along environmental gradients and among geographic locations separated with vast distance, particularly the debate of ‘Everything is everywhere’ and the importance of ‘the environment selects’ [25]. In this study, we focused on three geographically separated marine water columns: the SCS in western tropical North Pacific is characterized by permanent stratification and oligotrophic conditions; the CRD in eastern tropical North Pacific features a shallow thermocline (annual mean depth 35∼40 m) caused by upwelling and a permanent oxygen deficient zone between 400 and 700 m; while the subarctic site SNP is located in the Oyashio region of the western subarctic gyre where a spring phytoplankton bloom occurs annually. Those features mentioned above could support very different bacterial communities, however, the common physico-chemical environmental characteristics of the dark deep ocean, such as low temperature, low oxygen, less labile organic matters and high salinity existed among all the three regions would select similar bacterial populations. Therefore, it will be interesting to compare the bacterial composition and diversity in different water layers through the water columns and among these geographically distinctive locations. We notice that our samples were collected over a 5-year period. However, because all 3 sites are located in open ocean, no strong inter-annual variability is expected, though phytoplankton bloom in our mid-latitude site may affect bacterial composition in the surface layers [48].

### Diversity revealed from 454 pyrosequencing

The recent development of high throughput tag pyrosequencing methods has allowed recovering of much more detailed data about microbial diversity, abundance and community structure, thus offered an excellent opportunity to evaluate bacterial distribution profiles along various environmental gradients. Previous investigations of microbial community structure in the Pacific Ocean using conventional molecular techniques such as denaturing gradient gel electrophoresis (DGGE) and clone library likely underestimated both richness and diversity due to technical limitations. For example, based on 16S rRNA gene clone library, much lower number of OTUs (23∼59) and diversity (Shannon index of 2.51∼3.74) were detected from the eastern tropical North Pacific Ocean [26]; similar results were reported for bacterial communities in a lagoon in North Pacific Ocean [27]. However, about two orders of magnitude higher of both species richness (Chao1) and number of OTUs and two times higher of diversity index for bacterial communities had been detected with comparable coverage in our study by using pyrosequencing. In addition, the fact that more than 60 % of the total OTUs in our study were unique OTUs, which was in agreement with those reported from coastal environment to deep open ocean [28], further supports the point that microbial diversity was enhanced by the increased resolving power of new molecular tools and detailed community structures could be achieved in combination with fairly high taxonomic resolutions [16]. Even compared with other bacterial metagenomic studies from Arctic surface seawater [29], northern Baltic Sea [15], North Atlantic Ocean [17] and North Pacific Ocean [11], the diversity, richness and number of OTUs from our study were still significantly higher. This divergence could possibly be caused by different primers applied and different number of original reads generated. However, it is very unlikely as the North Atlantic Ocean study mentioned above had on average 18111 reads per sample [17], which is about 1.5 times of the reads per sample obtained in our study. Therefore the diversity revealed from pyrosequencing should not be affected by the original numbers of reads, but rather reflect the real bacterial community diversity.

### Spatial distribution of different bacterial groups

In the marine bacterial communities investigated here, a large proportion of the ribosomal sequences belonged to *Proteobacteria*, confirming their global distribution. *Proteobacteria* display a large phylogenetic diversity that may contribute to their successful colonization over a wide range of ecological niches. Our finding was consistent with previous phylogenetic studies on tropical Pacific Ocean using clone library [26], and confirmed their domination in the North Pacific Ocean, including tropical, subtropical and subarctic regions.

On the class level of *Proteobacteria*, *Alphaproteobacteria* predominated in all three regions, particularly the upper water column (surface and 100 m), comprising primarily free-living oligotrophic SAR11 representatives. It is well established that SAR11 dominates the surface waters globally [30]. *Gammaproteobacteria* was another dominant group of *Proteobacteria* in our study and their importance increased in deep waters. They were also reported as the most abundant group in the northern Baltic Sea [15]. The high abundance of *Gammaproteobacteria* detected in SNP is in agreement with Kataoka et al [31], who reported the predominance of *Gammaproteobacteria* in SNP based on estimation by DGGE.

*Cyanobacteria* represent 11∼20 % of the reads in the euphotic zone of all three locations, and the species composition was apparently different between the tropical and subarctic waters. GpIIa family including *Prochlorococcus* and *Synechococcus* were the most important *Cyanobacteria* in oligotrophic tropical surface water; this is well documented across the global oceans [32], [33], [34], [35]. On the other hand, majority of the *Cyanobacteria* in the subarctic waters came from the symbionts associated with *Bacillariophyta* and *Cryptomonadaceae* [36]. This result is supported by the results of Suzuki et al [37] reporting that diatoms and *Cryptophytes* were relatively abundant in SNP during the spring.

### Bacterial community variations along depths and among geographic locations

The existing of large gradient of physico-chemical conditions along the water depths, such as the decreasing in light, temperature and labile organic matter availability, have been identified as important impact factors affecting the vertical distribution of marine microbial communities [4], [30]. Physico-chemical parameters contributed almost equally in synergy to the bacterial vertical stratification in the NW Mediterranean Sea [38]. Among those abiotic factors, depth and latitude were the highly significant explanatory variables for bacterial populations from different water masses in the North Atlantic Ocean [17]. Microbial studies about depth effect had been frequently reported, however, studies covering different latitudes are still quite rare. In our study, oceanic stations from both tropical and subarctic regions of the Pacific Ocean were examined and strong difference in bacterial composition in the surface water between high latitude SNP and low latitude SCS and CRD was found, as indicated by the clear separation in both the NMDS plot and UPGMA dendrogram. This highlights the importance of horizontal spatial effects on the microbial community structures in the top layer of the ocean.

Regarding to the bacterial vertical profile alone, contradictory reports have been documented. Using fingerprinting techniques, bacterial richness and evenness in the deep waters were found as high and variable as those in the surface and subsurface waters in the North Pacific and Atlantic Oceans [39], as well as the eastern Mediterranean Sea [40]. However, after the application of the high throughput DNA pyrosequencing, decreased bacteria diversity with depth was found at the subtropical North Pacific station ALOHA [11], and the opposite pattern was discovered as well [20]. It may not be directly comparable between our work and some previous studies of bacterial assemblages over depth profiles in the Pacific Ocean that used other methodologies, such as clone-library [26] and metagenomics approach [4], or different primers that amplify domains of Archaea, Bacteria and Eucarya simultaneously [11].

In our study, higher bacterial species richness and diversity in the intermediate water columns than those from surface and deep waters were recovered from the geographically distinctive oceanic regions of SNP and CRD. Whether such vertical distribution pattern represents a general feature of the global ocean, and the mechanistic reasons that cause such pattern remain unexplained, but it is clear that the high numbers of unique OTUs, representing rare phylotypes, detected in the intermediate waters contribute largely to the in situ high diversity [19]. Both rarefaction curves and low coverage demonstrated that more sequencing efforts may be needed for all samples, though the diversity patterns might not be affected.

Our finding that the bacterial communities of the three regions shared high similarities (>80 %) in the deep waters, but were distinctive from upper layers, is consistent with the previous finding that deep-sea assemblages forming a separate cluster from surface assemblages in the North Pacific Ocean [11]. This may reflect the homogeneous nature of the generally DOC-poor deep ocean, contrasting to the highly spatially variable physico-chemical features in shallower waters. Echoing Koskinen et al's results in the northern Baltic Sea [15], which reported more uniform bacterial community horizontally at the same depth between different sites than vertically with the same water column, our findings provide evidence that even in the large scale of the whole Pacific Ocean the microbial community structure is affected by prevailing physical and hydrochemical conditions, rather than the geographic distance.

It is worthwhile to note that one sample in this study (CRD-500 m) is located in the oxygen minimum zone (OMZ) characterized by stably depleted oxygen concentrations (e.g. <20 µM). The co-occurrence of anaerobic and aerobic processes makes the OMZ a microbial hotspot, resulting in unique microbial assemblages and high microbial diversity [41], [42]. Our results also revealed higher species diversity in the OMZ than in both upper and lower oxic water layers (Table 1) and a distinctive community composition highly different to any other samples (Fig. 5). Also, CRD-500 m has the most (30%) unidentified sequences (Fig. 2a), reflecting the existence of large numbers of exotic microorganisms that are yet unknown. Although global OMZs occupy only a small portion (∼0.1 %) of the ocean, their role in affecting the biogeochemical cycles, especially the nitrogen cycle, has been noticed [43]. The release of ammonium and nitrite through incomplete denitrification and deficient dissolved oxygen in the OMZs support the growth of anammox bacteria, making OMZs an important environment for marine nitrogen loss. The detection of *Planctomycetes* in CRD-500 m in our study clearly supports the existence of anammox process in the OMZ of CRD.

Furthermore, many bacterial groups were found only in the deep layers, particularly the tropical waters. For example, *Moraxellaceae* and *Pseudoalteromonadaceae* were among the most abundant families in the 2000 m depth of SCS and CRD, respectively, and they contain mainly psychrotolerant bacteria that are able to live in extremely cold habitats such as deep sea [44], [45]. More detailed analysis is needed to reveal the identity and function of the vast unknown microorganisms in the deep ocean.

## Conclusion

Similar vertical variation patterns of Bacteria diversity were found in the three regions with different latitudes in the Pacific Ocean. Generally, bacterial community structures in the deep waters were quite different from those in upper water column, implying that the bacterial populations were affected by the prevailing physical and hydrochemical conditions along the water column. On the other hand, the bacterial community composition in the deep water of the three oceanic regions, which are thousands kilometers apart, were rather similar, supporting the old microbiological tenet ‘*Everything is everywhere, but, the environment selects*’ (Baas Becking 1934 [46], cited in de Wit and Bouvier 2006 [25]) as the environment of those deep sea is rather invariable. Given the highly spatial heterogeneity inherent to the marine environments and variations of microbial community structures resulted, a deep sequencing method such as pyrosequencing not only could provide exhaustive characterization of microbial assemblages, but would also be helpful to identify specific metabolic genes and gene expression, thus revealing the ecological roles of marine microbial communities [4], [47].
